# PTPIP51—A New RelA-tionship with the NFκB Signaling Pathway

**DOI:** 10.3390/biom5020485

**Published:** 2015-04-16

**Authors:** Alexander Brobeil, Fabian Kämmerer, Claudia Tag, Klaus Steger, Stefan Gattenlöhner, Monika Wimmer

**Affiliations:** 1 Institute of Anatomy and Cell Biology, Justus-Liebig-University, Gießen 35392, Germany; E-Mails: fabian.kaemmerer@med.uni-giessen.de (F.K.); claudia.tag@anatomie.med.uni-giessen.de (C.T.); monika.wimmer@anatomie.med.uni-giessen.de (M.W.); 2 Institute of Pathology, Justus-Liebig-University, Gießen 35392, Germany; E-Mail: stefan.gattenloehner@patho.med.uni-giessen.de; 3 Department of Urology and Pediatric Urology, Justus-Liebig-University, Gießen 35392, Germany; E-Mail: klaus.steger@chiru.med.uni-giessen.de

**Keywords:** PTPIP51, NFκB, RelA, p65 subunit

## Abstract

The present study shows a new connection of protein tyrosine phosphatase interacting protein 51 (PTPIP51) to the nuclear factor κB (NFκB) signalling pathway. PTPIP51 mRNA and protein expression is regulated by RelA. If bound to the PTPIP51 promoter, RelA repress the mRNA and protein expression of PTPIP51. The parallel treatment with pyrrolidine dithiocarbamate (PDTC) reversed the suppression of PTPIP51 protein expression induced by TNFα. Using the intensity correlation analysis PTPIP51 verified a co-localization with RelA, which is also regulated by TNFα administration. Moreover, the direct interaction of PTPIP51 and RelA was established using the DuoLink proximity ligation assay. IκBα, the known inhibitor of RelA, also interacted with PTPIP51. This hints to the fact that in un-stimulated conditions PTPIP51 forms a complex with RelA and IκBα. The PTPIP51/RelA/IκBα complex is modulated by TNFα. Interestingly, the impact on the mitogen activated protein kinase pathway was negligible except in highest TNFα concentration. Here, PTPIP51 and Raf-1 interactions were slightly repressed. The newly established relationship of PTPIP51 and the NFκB signaling pathway provides the basis for a possible therapeutic impact.

## 1. Introduction

In recent studies PTPIP51 was identified to be strongly involved in the mitogen activated kinase (MAPK) signaling [1,2]. Here, PTPIP51 is under control of distinct kinases and phosphatases modulating its binding capability to Raf-1 and thereby titrating the MAPK signal [1,2,3]. Moreover, the protein interactions of PTPIP51 are associated with cell cycle progression and PTPIP51 plays a pivotal role during chromosome segregation [4].

Recently, De Vos and co-workers corroborated the binding of PTPIP51 to the outer layer of mitochondria, where PTPIP51 function was linked to processes in the calcium homeostasis by interacting with vesicle associated protein B (VAPB) [5]. Yet, the first functional study to characterize the cellular implications of PTPIP51 showed that PTPIP51 is linked to the apoptotic process [6]. In HEK293T and HeLa cells, the full-length transcript of PTPIP51 exhibits a co-localization with mitochondria and is anchored by its transmembrane domain at the outer layer of mitochondria. Of note, if overexpressed in these cell lines, PTPIP51 induces apoptosis with all hallmarks—PARP and caspase-3 cleavage, shrinkage of the cell and fragmentation of the nucleus [6].

However, the apoptotic function of PTIP51 and its associated signaling pathways remain undissolved. The tumor necrosis factor alpha (TNFα) is linked to apoptosis in various physiological somatic and malignant transformed cells [7,8,9,10]. Moreover, the TNFα signal is coupled to a variety of downstream effector pathways, such as the endoplasmatic reticulum apoptosis pathway via EGFR-PI3K and the p38 MAPK pathway [9,11]. The latter induces apoptosis in glioma cells [11]. Of note, in contrary to normal neuronal tissue PTPIP51 is expressed in transformed malignant glioma cells [12].

The TNFα induced apoptosis is limited and antagonized by the activation of the transcription factor nuclear factor kappa B (NFκB). Followed by TNF receptor I activation formation of two complexes occurs. The Complex I is formed by TNF-R1, TRADD, RIP, TRAF2 and c-IAP1 at the cell membrane, which in turn promotes the apoptotic function mediated by TNFα. The Complex II consists of FADD and pro-caspases 8/10 located in the cytosol and subsequently activates the NFκB pathway for cell survival [13]. Interestingly, the complex II only can induce apoptosis if NFκB fails to up-regulate anti-apoptotic proteins [13]. The NFκB transcription factor family comprises five proteins, p65 (RelA), RelB, c-Rel, p105/p50 (NF-κB1), and p100/52 (NF-κB2) [14]. Notably, PTPIP51 possesses a promoter binding site for RelA as annotated in the USSC genome browser [14] Moreover, p65 (RelA) interacts with protein kinase A (PKA) and is enhanced in its transcriptional regulation by serine phosphorylation [15]. PKA also interacts with PTPIP51 facilitating its binding to Raf-1 [1].

Constitutive activation of NFκB is a factor rendering cancer cells resistant to chemotherapy resulting in uncontrolled cell proliferation [16]. This is seen in glioma cells where the expression of NFkB leads to uncontrolled proliferation [17]. As known PTPIP51 is also expressed in glioma cell [12]. Targeting the protein-protein interaction PTPIP51/RelA and PTPIP51/IκBα may be an option for alternative chemotherapies. Such alternative therapeutic options are demanded because of the failure of established drugs as recently shown for bevacizumab in recurrent glioma [18].

Therefore, we investigated the human keratinocyte cell line HaCaT for the effect of TNFα administration to the PTPIP51 protein expression. In addition, the possible regulation of PTPIP51 gene transcription was assayed by qualitative polymerase chain reaction. The duolink proximity ligation assay was used for displaying possible interactions of PTPIP51 and members of the NFκB family, especially with p65 (RelA). Here, we established two new interaction partners of PTPIP51: RelA and IκBα. Furthermore, PTPIP51 mRNA and protein expression were regulated by the administration of TNFα.

## 2. Results

### 2.1. The PTPIP51 and RelA mRNA Expression is Regulated by TNFα Administration

**Figure 1:**
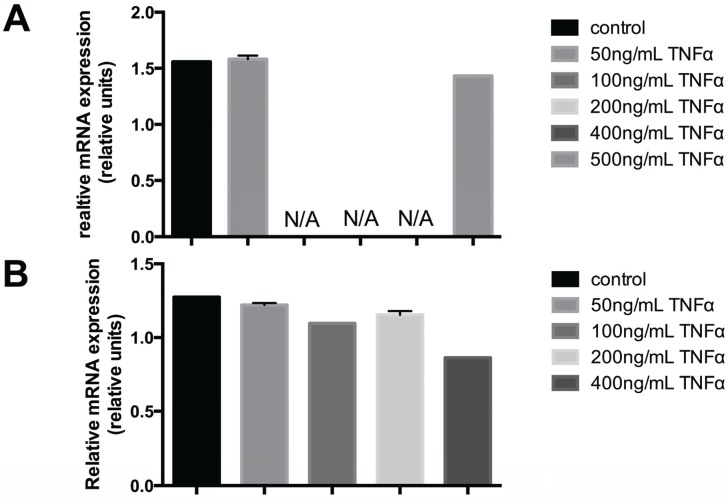
(**A**) PTPIP51 mRNA expression in untreated human keratinocytes and in TNFα treated cells (50 ng/mL, 500 ng/mL). The expression is given in relative units. N/A: not measurable. (**B**) RelA mRNA expression in untreated human keratinocytes and in TNFα treated cells (50ng/mL, 100ng/mL, 200ng/mL, 500ng/mL).

Quantitative polymerase chain reaction experiments displayed PTPIP51 mRNA expression to be regulated by TNFα administration. If exposed to increasing TNFα concentrations PTPIP51 expression showed a sharp drop at 100 ng TNFα. Using concentrations of 200 and 400 ng/mL TNFα there was no traceable PTPIP51 mRNA (Figure 1A). Noteworthy, application of 500 ng/mL TNFα restored near normal values of PTPIP51mRNA. Analyzing the RelA mRNA expression under TNFα treatment, displayed a concentration dependent decline in mRNA concentration (Figure 1B).

### 2.2. The PTPIP51 Protein Expression is also Regulated by TNFα

**Figure 2:**
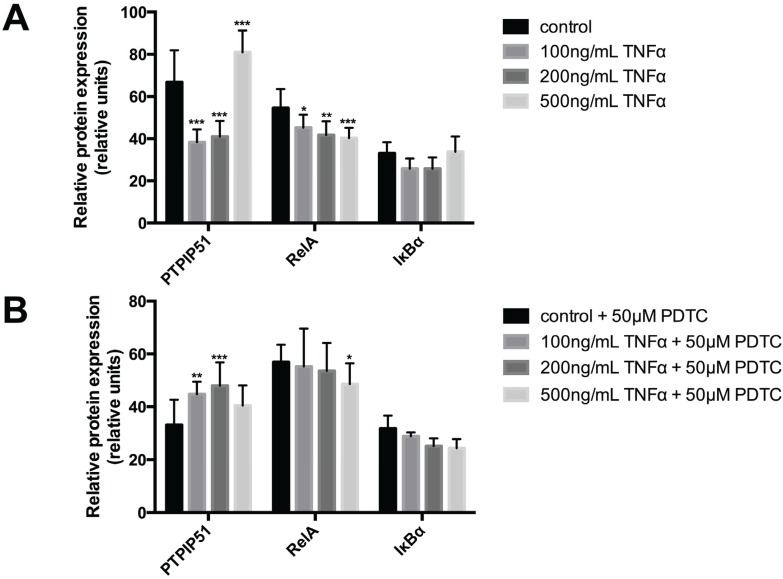
Semiquantitative expression of PTPIP51, RelA and IκB protein in keratinocytes. (**A**) PTPIP51 protein (left panel), RelA (middle panel) and in IκB (right panel) in untreated controls, 100 ng/mL TNFα treated cells, 200 ng/mL TNFα treated cells, 500 ng/mL TNFα treated cells. * (*p* < 0.05), ** (*p* < 0.01), *** (*p* < 0.001); (**B**) PTPIP51 protein (left panel), RelA (middle panel) and IκB (right panel) in controls treated with 50 µM PDTC, 100 ng/mL TNFα and 50 µM PDTC treated cells, 200 ng/mL TNFα and 50 µM PDTC treated cells, 500 ng/mL TNFα and 50 µM PDTC treated cells. * (*p* < 0.05), ** (*p* < 0.01), *** (*p* < 0.001). The resulting data were analyzed by GraphPad Prism 6 software (GraphPad Software, La Jolla, CA, USA), significance of results tested by Dunnett’s multiple comparisons test.

The protein expression of PTPIP51 in the HaCaT cell line was investigated by a semi quantitative approach using Image J. PTPIP51 protein expression was significantly decreased at levels of 100 ng and 200 ng/mL TNFα (Figure 2A). Noteworthy, treatment with 500 ng/mL TNFα led to supranormal PTPIP51 protein levels (Figure 2A). The differences between the untreated control group and all used TNFα concentrations were highly statistically significant (*** *p* < 0.001). RelA showed a continuous decrease in protein expression levels with increasing TNFα concentrations (Figure 2A). Compared to the control value, the differences were statistically significant. IκBα expression displayed no significant reduction by TNFα treatment (Figure 2A).

This effect was retracted for PTPIP51 as well as RelA by the administration of pyrrolidine dithiocarbamate (PDTC), an inhibitor of NFκB activation, (Figure 2B). PTPIP51 protein expression was elevated by 100 ng/mL TNFα combined with 50 µM PDTC (** *p* < 0.01) and was further raised by the treatment with 200 ng/mL TNFα in combination with 50 µM PDTC (*** *p* < 0.001). 500 ng/mL TNFα combined with 50 µM PDTC slightly reduced PTPIP51 protein, yet with levels still higher than those observed for cells submitted only to PDTC (*p* > 0.05) (Figure 2B). RelA protein continuously decreased with all investigated TNFα concentrations in combination with PDTC (50 µM), but to a lesser degree than by sole application of TNFα (Figure 2B). IκBα protein also was progressively reduced by increasing TNFα concentrations despite their combination with 50 µM PDTC (Figure 2B).

### 2.3. PTPIP51 is co-Localized with RelA in the HaCaT Cell Line and the co-Localization is Altered by TNFα

**Figure 3:**
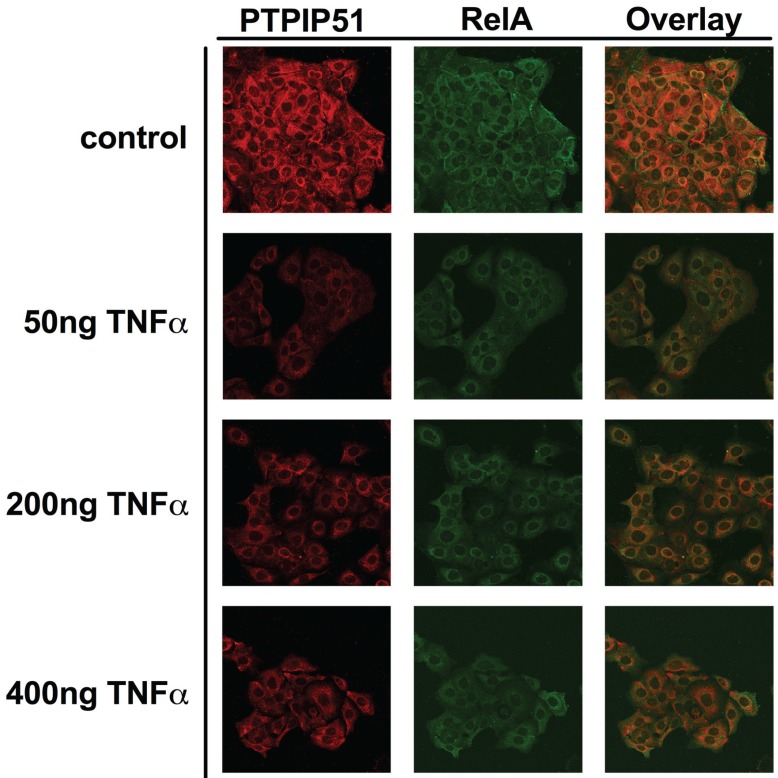
Immunocytochemical staining of PTPIP51 and RelA in human keratinocytes. Upper panel: untreated controls: PTPIP5, RelA, overlay. Second panel: 50 ng/mL TNFα treated cells: PTPIP51, RelA, overlay. Third panel: 200 ng/mL TNFα treated cells: PTPIP51, RelA, overlay. Fourth panel: 400 ng/mL TNFα treated cells: PTPIP51, RelA, overlay. Co-localization is indicated by organge colour.

Confocal laser scanning microscopy experiments displayed a co-localization of PTPIP51 with RelA (Figure 3 first row). The co-localization is indicated by the orange color in the overlayed PTPIP51 and RelA confocal images (Figure 3 right row Overlay).

**Figure 4:**
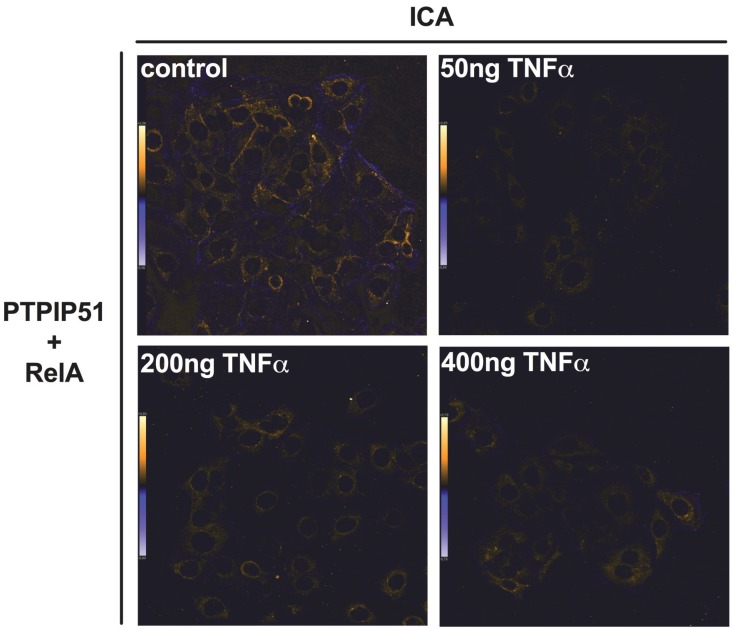
Intensity correlation (ICA) of PTPIP51 and RelA. ICA was determined for PTPIP51 and RelA in untreated controls, 100 ng/mL TNFα treated cells, 200 ng/mL TNFα treated cells, 400 ng/mL TNFα treated cells. The co-localization of PTPIP51 and RelA is displayed in orange. Sites of non-co-localization are marked in blue.

This co-localization was corroborated by the intensity correlation analysis. The calculated co-localization by ICA, basing on the comparison of fluorescence intensities (see Materials and Methods), is displayed in Figure 4. The co-localization is indicated in yellow to orange and parts with non-co-localization are shown in blue. Administrating 50 ng of TNFα resulted in the dissociation of PTPIP51 and RelA as shown in Figure 4. The co-localization was partially restored at 200 and 500 ng of TNFα (Figure 4).

### 2.4. PTPIP51 Interacts with RelA in HaCaT Cells

**Figure 5:**
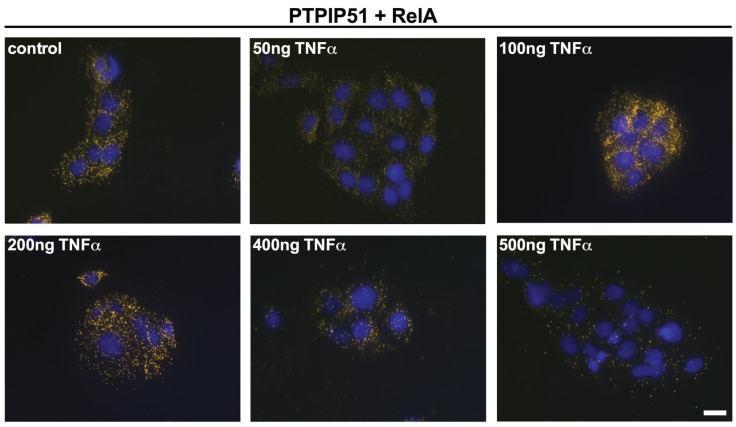
Interactions of PTPIP51 and RelA in human keratinocytes determined by Duolink proximity assay. Untreated controls, 50 ng/mL TNFα treated cells, 100 ng/mL TNFα treated cells, 200 ng/mL TNFα treated cells, 400 ng/mL TNFα treated cells, 500 ng/mL TNFα treated cells. Bar: 20 µm.

The interactions of PTPIP51 were analyzed by Duolink Proximity ligation assay. As seen in Figure 5 PTPIP51 interacts with RelA. This interaction is regulated by TNFα. High concentration (400 ng and 500 ng) reduced the number of interactions per cell.

**Figure 6:**
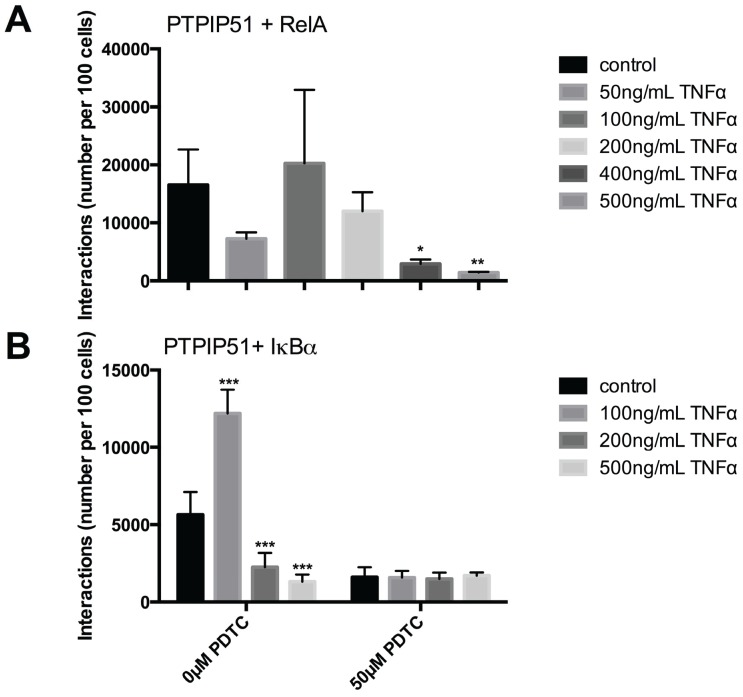
Quantitative analysis of PTPIP51 and RelA and PTPIP51 and IkBα interactions evaluated by Duolink Image Tool software. (**A**) PTPIP51 and RelA interactions in human keratinocytes. Untreated controls, 50 ng/mL TNFα treated cells, 100 ng/mL TNFα treated cells, 200 ng/mL TNFα treated cells, 400 ng/mL TNFα treated cells, 500 ng/mL TNFα treated cells. * (*p* < 0.05), ** (*p* < 0.01); (**B**) PTPIP51 and IkBα interactions. Left panel: Untreated controls, 50 ng/mL TNFα treated cells, 100 ng/mL TNFα treated cells, 200 ng/mL TNFα treated cells, 400 ng/mL TNFα treated cells, 500 ng/mL TNFα treated cells. *** (*p* < 0.001). Right panel: controls, 50 ng/mL TNFα treated cells, 100 ng/mL TNFα treated cells, 200 ng/mL TNFα treated cells, 400 ng/mL TNFα treated cells, 500 ng/mL TNFα treated cells. All cells were treated with 50 µM PDTC. The resulting data were analyzed by GraphPad Prism 6 software, significance of results tested by Dunnett’s multiple comparisons test.

Quantification of these interactions was performed by the Duolink Image Tool software and subsequent statistical analysis (Figure 6A). The analysis revealed a biphasic profile with a sharp statistically significant reduction by 50 ng TNFα treatment and an increase when TNFα is increased to 100 ng, reaching supra control values. Further augmentation of TNFα resulted in a continuous decrease of the PTPIP51/RelA interactions.

### 2.5. PTPIP51 Interacts with IκBα in HaCaT Cells

**Figure 7:**
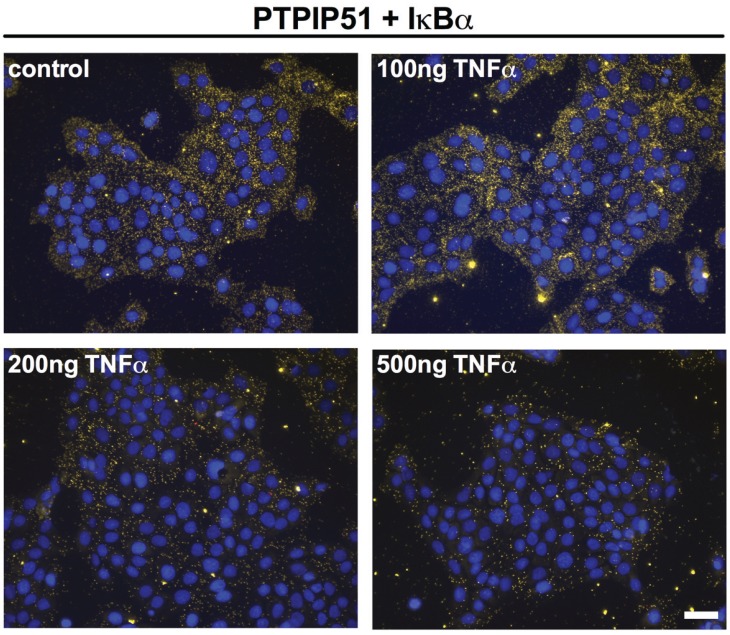
Interactions of PTPIP51 and IkBα in human keratinocytes determined by Duolink proximity assay. Untreated controls, 100 ng/mL TNFα treated cells, 200 ng/mL TNFα treated cells, 500 ng/mL TNFα treated cells. Bar: 20 µm.

IκBα inhibits NF-κB by keeping it in a cytoplasmic localization thus preventing its action as a transcription factor. Testing a probable interaction of PTPIP51 with IκBα evidenced an active interaction profile, which was also regulated by TNFα (Figure 7).

Quantification of these interactions was performed by the Duolink Image Tool software and subsequent statistical analysis (Figure 6B). The analysis revealed a profile with a highly significant (*** *p* < 0.001) increase in interactions by 100 ng TNFα and a sharp reduction by 200 and 500 ng, which was highly statistically significant (*** *p* < 0.001) (Figure 6B). The additional application of PDTC had no effect on PTPIP51/IκBα under the influence of TNFα (*p* > 0.05). Yet, compared to the completely untreated control cells PDTC drastically lowered the PTPIP51/IκBα interaction to approximately one third (Figure 6B).

### 2.6. PTPIP51 Interacts with 14-3-3, Raf-1, MEK1 in HaCaT Cells

**Figure 8:**
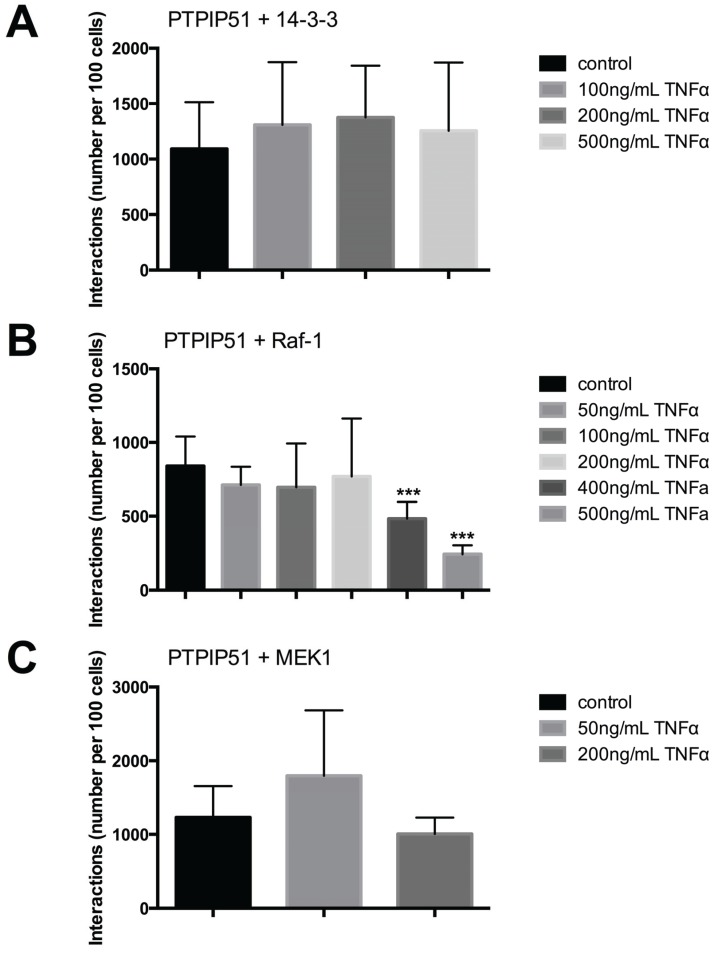
Quantitative analysis of PTPIP51 and 14-3-3, PTPIP51 and Raf-1, PTPIP51 and MEK1 interactions evaluated by Duolink Image Tool software. (**A**) PTPIP51 and 14-3-3 interactions in human keratinocytes. Untreated controls, 50 ng/mL TNFα treated cells, 100 ng/mL TNFα treated cells, 200 ng/mL TNFα treated cells, 400 ng/mL TNFα treated cells, 500 ng/mL TNFα treated cells; (**B**) PTPIP51 and Raf-1 interactions. Untreated controls, 50 ng/mL TNFα treated cells, 100 ng/mL TNFα treated cells, 200 ng/mL TNFα treated cells, 400 ng/mL TNFα treated cells, 500 ng/mL TNFα treated cells. *** (*p* < 0.001); (**C**) PTPIP51 and MEK1 interactions. Untreated controls, 50 ng/mL TNFα treated cells, 100 ng/mL TNFα treated cells, 200 ng/mL TNFα treated cells, 400 ng/mL TNFα treated cells, 500 ng/mL TNFα treated cells. The resulting data were analyzed by GraphPad Prism 6 software, significance of results tested by Dunnett’s multiple comparisons test.

The TNFα regulation of PTPIP51 involvement in MAPK signaling was investigated by the analysis of PTPIP51 interactions with members of MAPK pathway. The interactions of PTPIP51 with 14-3-3 were slightly increased by the application of increasing TNFα concentrations. In relation to controls there were no statistically significant differences (*p* > 0.05) (Figure 8A).

In contrast the PTPIP51/Raf-1 interactions were regulated by high concentrations of TNFα. Highly statistically significant differences to controls were seen for 400 ng and 500 ng TNFα (*** *p* < 0.001) (Figure 8B).

The PTPIP51/MEK1 interactions were increased by low TNFα (50 ng) and decreased by 200 ng TNFα (Figure 8C).

### 2.7. PTPIP51 Interacts with PKA, PTP1B in HaCaT Cells

**Figure 9:**
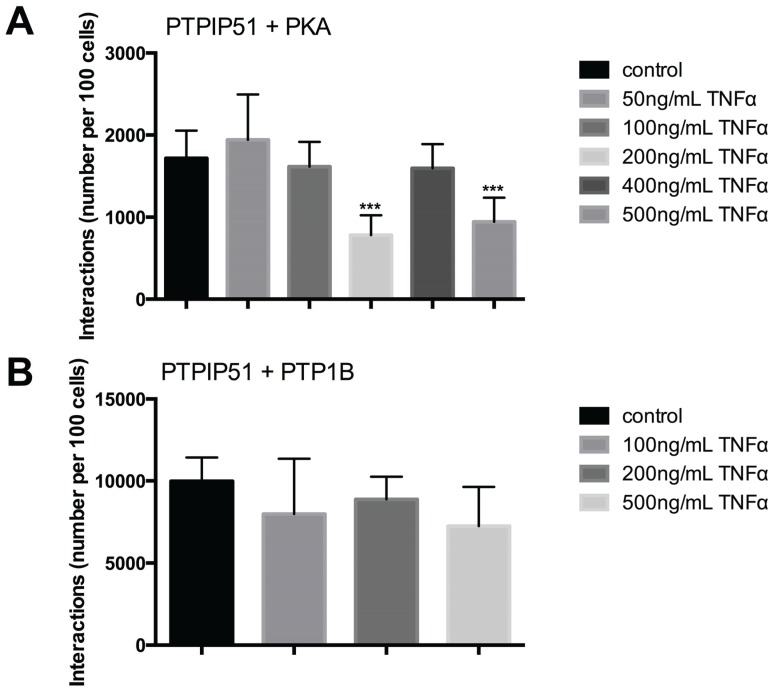
Quantitative analysis of PTPIP51 and PKA, PTPIP51 and PTP1B interactions evaluated by Duolink Image Tool software. (**A**) PTPIP51 and PKA interactions. Untreated controls, 50 ng/mL TNFα treated cells, 100 ng/mL TNFα treated cells, 200 ng/mL TNFα treated cells, 400 ng/mL TNFα treated cells, 500 ng/mL TNFα treated cells. *** (*p* < 0.001); (**B**) PTPIP51 and PTP1B interactions. Untreated controls, 50 ng/mL TNFα treated cells, 100 ng/mL TNFα treated cells, 200 ng/mL TNFα treated cells, 400 ng/mL TNFα treated cells, 500 ng/mL TNFα treated cells. The resulting data were analyzed by GraphPad Prism 6 software, significance of results tested by Dunnett’s multiple comparisons test.

The interactions of PTPIP51 are regulated by its serine phosphorylation status regulated by Protein kinase A. Thus, we investigated the interaction of PTPIP51 with PKA in HaCaT cells submitted to TNFα in increasing concentrations. 50 ng and 100 ng TNFα had no significant influence on PTPIP51/PKA interaction. Using 200 ng TNFα significantly reduced the interaction, whereas 400 ng led to near normal values and 500 ng restored the low interaction rate seen with 200 ng (Figure 9A).

The tyrosine phosphorylation status also regulates the interaction profile of PTPIP51. PTP1B is responsible for the dephosphorylation of Tyr176, which in its phosphorylated state inhibits the interactions of PTPP51. Therefore, we analyzed the interaction profile of PTPIP51 and PTP1B und the influence of increasing concentrations of TNFα. The quantity of interactions was reduced by all TNFα concentrations but the reductions were not statistically significant (*p* > 0.05) (Figure 9B).

## 3. Discussion

PTPIP51 protein expression is regulated by the epidermal growth factor (EGF), transforming growth factor beta (TGF-β), retinoic acid (RA) and vitamin D3 [18]. HaCat cells incubated with either RA or vitamin D3 display a concentration dependent increase of PTPIP51 positive cells [19]. In contrast, applying low EGF to HaCaT cells results in a decrease of PTPIP51 positive cells accompanied by a stepwise up-regulation of PTPIP51 protein expression using higher EGF concentrations [19].

TNFα treatment of HaCaT cells showed similar results as obtained for EGF administration. Both PTPIP51 mRNA expression as well as PTPIP51 protein expression is decreased in HaCaT cells after application of TNFα in a concentration range from 100 ng/mL to 400 ng/mL. Interestingly, using 500 ng/mL TNFα neither affects PTPIP51 mRNA expression nor protein expression. Moreover, PTPIP51 protein expression slightly exceeded values of untreated HaCat cells. Remarkably, PTPIP51 exhibits a RelA binding site at its promoter region (UCSC genome browser) [14].

RelA forms a complex with IκBα, the inhibitory protein of RelA [20]. After a transient TNF-α stimulation RelA initiates resynthesis of IκBα as a potent negative feedback resulting in the rapid termination of RelA activity [20]. Data given here where IκBα protein displays no significant changes by TNFα treatment corroborate this observation. Moreover, RelA is degraded by the proteasome to terminate the RelA transcriptional activity [20].

NFkB activation can result in either cell survival or apoptosis induction, determined by the activating event [21]. RelA is able to induce transcription of anti-apoptotic proteins [22]. Yet, RelA is also able to repress transcription of anti-apoptotic proteins, when activated by ultraviolet light or by the chemotherapeutic drug daunorubicin/doxorubicin [22]. PTPIP51 was identified as a pro-apoptotic protein by binding to the mitochondria with its transmembrane domain [6]. The binding breaks down the membrane potential of the mitochondria with subsequent cytochrome-C release [6]. Of note, RelA is uniquely able to repress PTEN, a negative regulator of the PI3K/Akt pathway promoting cell survival [23]. Thus, RelA may suppress PTPIP51 expression level to maintain cell survival. When overexpressed PTPIP51 initiates apoptosis in the HEK293T and HeLa cell lines. Therefore, the down-regulation of PTPIP51 by RelA goes along with the cell survival protection of RelA [23].

The current study established two additional interaction partners of PTPIP51, namely RelA and IκBα. When complexed by IκBα, RelA is inhibited to translocate to the nucleus and to initiate transcription of target genes [24]. The IκB protein family covers the nuclear translocation signal (NLS) of RelA [24]. Interestingly, PTPIP51 also possesses a NLS [25], enabling its possible translocation to the nucleus. PTPIP51 interacted with RelA and IκBα in a dose dependent manner. The interaction of PTPIP51 with either RelA or IκBα was decreased for all TNFα concentrations except at 100 ng/mL resulting in an increase of both interactions. Thus, we presume that PTPIP51 is part of the inhibited RelA complex. In this setting PTPIP51 may cover additional sites of RelA essential for binding partners of RelA. Next to the interaction with IκBα RelA forms heterodimers with another NFκB family member, namely p50 the active form of NFκB1/p105 [26]. Here, PTPIP51 may inhibit the formation of the heterodimer preventing the activation of the canonical NFκB pathway. This is paralleled along with the similar interaction behavior of PTPIP51 with RelA as wells as with IκBα.

Besides the transcriptional regulation of target genes RelA is linked to other signaling pathways. During oxidative stress in myoblasts RelA is phosphorylated at its serine 276 residue by MSK1 [27]. Interestingly, MSK1 is a downstream signaling molecule of the MAPK pathway [27]. Yet, the cross-talk of the MAPK pathway with the NFκB pathway does not alter the translocation of RelA to the nucleus, but a fine titration of the RelA action is assumed [27]. PTPIP51 is linked to both pathways by interacting with Raf-1 through the scaffold protein 14-3-3 [3] and additionally interacts with RelA. Thus, it is of utmost interest whether PTPIP51 interaction levels are altered by the administration of TNFα and the subsequent dissociation of the PTPIP51/RelA/IκBα complex. Of note, in radioresistant MCF-7 breast cancer cells, RelA is able to inhibit the MAPK pathway. Increased levels of RelA were associated with a decrease in mitogen-activated protein kinase (MEK) and extracellular signal-regulated kinase (ERK) phosphorylation levels [28]. RelA interacts with MEK as well as with ERK, inhibiting the latter if complexed to translocate to the nucleus. Thus, we investigated the PTPI51/Raf-1 and the PTPIP51/MEK interactions. TNFα concentration ranging from 50 ng/mL to 200 ng/mL did not affect PTPIP51/Raf-1 interaction levels, whereas 400 and 500 ng/mL TNFα led to a decrease. This goes along with the observed MAPK inhibition of radioresistant MCF-7 cells displaying a MAPK inhibition if RelA is over-activated [28]. Interestingly, the PTPIP51/MEK was also unchanged in lower concentrations (50–200 ng/mL) of TNFα. Moreover, the interaction with its scaffold protein was completely unaffected by TNFα administration. Malignant diseases, such as the Hodgkin lymphoma express high levels of RelA in both Hodgkin and Reed-Sternberg cells. Our results can help to clarify probable dysregulations in the PTPIP51/RelA/IκBα axis affecting the MAPK pathway [29]. Interestingly, termination of the sustained NFκB activation leads to apoptosis in Hodgkin and Reed-Sternberg cells [29]. Up to now, the chemotherapeutic regime for the Hodgkin lymphoma consists of a combination of seven substances, namely bleomycin, etoposide, doxorubicin, cyclophosphamide, vincristine, procarbazine, and prednisone [30], increasing the risk of young patients to develop secondary malignancies [31]. Therefore, it is of utmost interest to identify alternative drugs decreasing cytotoxity of chemotherapies. The newly established interaction of PTPIP51 and RelA may resemble a drugable interaction for more specific anticancer therapy. PKA also enhances the binding of PTPIP51 to Raf-1 and in consequence the activation of the MAPK pathway [1,2,32]. This effect is probably mediated by phosphorylating the serine 212 residue of PTPIP51 [1,25]. Notably, p65 (RelA) also interacts PKA enhancing its transcriptional regulation by serine phosphorylation [15]. The HaCat cell line exhibited a reduced PTPIP51/PKA interaction levels when incubated with either 200 ng/mL or 500 ng/mL TNFα, respectively. All other concentration had no effect on this interaction. This may be a possible new regulation mechanism for titrating the MAPK activation through the crosstalk with the NFκB signaling pathway. Interestingly, the interaction with the relevant tyrosine phosphatase PTP1B was unaffected by the administration of TNFα. Therefore, we conclude that PTP1B exerts its regulatory function on PTPIP51/Raf-1 binding levels when the main tyrosine receptor kinases are activated, such as the epidermal growth factor rector (EGFR) [2,4]. PKA is assumed to titrate the activation of PTPIP51/RelA mediated MAPK pathway activation.

## 4. Experimental Section

### 4.1. Cell Culture

All experiments were performed with HaCaT cells kindly provided by Dr. Hansjörg Teschemacher (Department of Pharmacology, Justus Liebig University, Giessen, Germany) with the permission of Dr. Norbert Fusenig (DKFZ, Heidelberg, Germany, MTA number L-4598).

Cells were kept at 37 °C in humidified 5% CO2 atmosphere and were cultured in Roswell Park Memorial Institute (RPMI) 1640 medium (PAA, Paching, Austria, Cat.# E15-840) supplemented with 10% fetal calf serum (FCS), 100 U/mL penicillin, 100 µg streptomycin. For inhibition experiments cells were grown on culture slides coated with FCS. Cells were grown until near confluence was reached. Subsequently, the medium was removed and the cells were treated with the inhibitors given in Table 1 for 1 h. In case of EGF stimulation the cells were pre-incubated with the PBS/Glucose solution for 1 h. Subsequently the inhibitors supplemented with 7–10 M EGF were applied for 1 h. Both reactions were terminated by withdrawing the medium supplemented with the inhibitor, adding ice cold PBS and fixation of the cells with 4% paraformaldehyde.

**Table 1:** List of antibodies used for the immunocytochemistry and DPLA.

Antibody	Immunogen	Antibody Source	Clone	Dilution	Manufacturer
PTPIP51 (P51ab)	Human recombinant PTPIP51 protein encoding amino acids (aa) 131–470	Rabbit polyclonal		1:500	Prof. H. W. Hofer, Biochemical Department, University Konstanz, Germany
RelA	Peptide corresponding to human p65 coupled to BSA	Mouse monoclonal	12H11	1:100	Merck Millipore, Schwalbach, Germany Cat.# MAB3026
PTP1B	Recombinant protein corresponding to aa 1–321 of human PTP1B	Mouse monoclonal	107AT531	1:200	Abnova, Taipei, Taiwan Cat.# MAB1152
Raf-1	Mapping the C-terminus of Raf-1	Mouse monoclonal	E-10	1:100	Santa Cruz Biotechnology, Dallas, TX, USA Cat.# sc-7267
PKA	Recombinant fragment corresponding to aa 1–121 of human PKA	Mouse monoclonal	N/A	1:100	Abcam, Cambridge, UK Cat.#ab58187
IκBα	Recombinant Human IκB alpha/NFKBIA protein 02	Mouse monoclonal	MM02	1:100	Sino Biological Inc., North Wales, PA, USA Cat.# 12045-H07E
14-3-3beta	Specfic for an epitope mapping between aa 220–244 at the C-terminus of 14-3-3β of human origin	Mouse monoclonal	A-6	1:100	Santa Cruz Biotechnology, Dallas, TX, USA Cat.#sc-25276
MEK1	Purified MEK from human T cells and recombinant MEK1	Mouse monoclonal	3D9	1:100	Life Technologies, Carlsbad, CA, USA Cat.# 13-3500

HaCaT cells for interaction experiments were kept under same conditions as described above. Untreated and TNFαlpha treated cells were grown on culture slides coated with FCS. Cells were subsequently fixed with methanol and either immunocytochemistry or Duolink Proximity assay were applied.

### 4.2. TNFα Treatment

HaCaT cells were incubated with TNFα (Recombinant Human TNF-α, Peprotech Germany, Hamburg Germany, Cat.# 300-01A) in different concentrations (50–500 ng/mL) for 6 h at 37 °C. The reaction was stopped by the addition of methanol for fixing the cells or by transferring the cells either into RNA later (for mRNA determination) or into Laemmli buffer (for immunoblotting).

PDTC treatment: HaCaT cells were incubated with Ammonium pyrrolidine dithiocarbamate (Sigma-Aldrich, Cat.# P 8765, Munich, Germany) in a concentration of 50 µM for 6 h at 37 °C.

### 4.3. Quantitative Real Time PCR

The cells were transferred into RNA-later (Qiagen, Hilden, Germany) and stored deep frozen at −20 °C according to the manufacturer’s instructions. The RNA extraction was performed using the RNA extraction kit RNeasy MINI (Qiagen) according to the manufacturer’s instructions.

The amplification of cDNA was carried out in 25 μL reaction volume on the iCycler iQ Real-Time PCR Detection System (Bio Rad, Munich, Germany). The final reaction tubes contained 100 nM as PTPIP51 (forward primer: 5'-TCCAAGTGCTACAGAGAACTAGGGA-3' reverse primer: 5'-CCTCCAGAGCTTCCTAAAGGCTGA-3', RelA (forward primer: 5'-AAGAAGGGACCTGGAG-3'; reverse primer: 5'-CGCACTGTCACCTGGAAG-3') and reference gene ß-actin, 12.5 μL iQ SYBR Green Supermix (Bio Rad) and 2 μL of DNA template. The PCR conditions were 94 °C for 3 min followed by 40 cycles for 30 s, 60 °C for 30 s and 72 °C for 1 min. Melting curves were generated for all three genes after amplification. Negative controls were included in each run. While amplification of a 90 bp ß-actin product served as positive control, negative controls included samples lacking reverse transcriptase. The Ct values of each investigated gene were exported by the BioRad iCycler software. mRNA expression levels of PTPIP51 gene (target gene) was normalized to the expression of the house keeping gene β-actin. Results were visualized using GraphPad Prism 6 statistical software.

### 4.4. PTPIP51 Antibody (aa 131–470)

PTPIP51 antibody (Table 1) were produced as described previously [33,34].

### 4.5. Immunohistochemistry

Immunohistochemistry was performed as previously described by Koch *et al.* [33]. Prior to immunostaining nonspecific binding sites were blocked with 0.1 M phosphate buffered saline (PBS, pH 7.4) containing 5% bovine serum albumin and 5% normal goat serum. Samples were incubated overnight at room temperature with primary antibodies (see Table 1) diluted in PBS, followed by washing in PBS (three times for 10 min) and subsequent incubation for 1 h at room temperature with the respective secondary antibodies (see Table 1). The slides were washed in PBS, coverslipped in carbonate buffered glycerol at pH 8.6 and evaluated either by epifluorescence microscopy or by sequential confocal laser scanning microscopy.

PTPIP51 (aa 131–470) and peptide specific PTPIP51 antibodies were visualized either by Alexa Fluor 555 secondary antibody or Cy3-anti guinea pig antibody. Anti-mouse antibodies used for double staining were visualized by using Alexa Fluor 488 secondary antibody. Nuclei were displayed through Dapi.

### 4.6. Epifluorescence Microscopy

The Axioplan 2 fluorescence microscope equipped with Plan-Apochromat objectives (Carl Zeiss Jena, Jena, Germany) was used for photo documentation. For visualization of the secondary antibody Alexa Fluor 555 an excitation filter with a spectrum of 530–560 nm and an emission filter with a spectrum 572–647 nm were used. Alexa Fluor 488 was visualized by an excitation filter with a range of 460–500 nm and an emission filter with a range of 512–542 nm.

### 4.7. Confocal Laser Scanning Microscopy

Confocal Laser Scanning Microscopy Confocal images of cells were obtained with a Leica confocal laser scanning microscope (CLSM, TCS SP2, Leica, Bensheim, Germany). Confocal images of Cy3 fluorescence were acquired using Plan-Apochromat × 63/1.4 oil objective, 548 nm excitation wavelengths (helium-neon laser) and a 560–585 nm bandpass filter. The pinhole diameter was set to yield optical sections of 1 Airy unit. For the detection of Alexa 488, we used a Plan-Apochromat × 63/1.4 oil objective, the 488 nm excitation wavelength of an argon laser, and a 505–530 nm band-pass filter. The pinhole diameter was set to yield optical sections of 1 Airy unit. Confocal images were exported from the Leica software and stored as TIFF files. Image brightness and contrast were adjusted. Acquired images were subsequently processed by ImageJ (v1.43m; Rasband, W.S., ImageJ, U.S. National Institutes of Health, Bethesda, Maryland, USA, 1997–2011) using an iterative deconvolution plug-in by Bob Dougherty. Options were set for all confocal acquired images as follows: 8—numbers of iteration; and 2.0 pixels—LP filter diameter. Point spread function was calculated for each channel separately by the ImageJ plug-in created by Bob Dougherty [4].

Intensity Correlation Analysis: Intensity correlation analysis (ICA) was carried out using ImageJ (v1.43m) and an appropriate plug-in for ICA included in the plug-in package of the Wright cell imaging facility [4].

### 4.8. Semiquantification of Immunofluorescence Intensities

For semiquantifying immunfluorescence intensities, immunostaining intensities of HaCaT cells were registered for encircled single cells with the ImageJ program. Intensities of 50 cells/group were measured. The results of PTPIP51 immunofluorescence were plotted in a diagram by Graphpad Prism 6.

### 4.9. Duolink Proximity Ligation Assay (DPLA)

*In situ* interactions were detected by the proximity ligation assay kit Duolink II (PLA probe anti-rabbit minus, Cat.# 92005, PLA probe anti-mouse plus, Cat.# 92001; Dection Kit Orange, Cat.# 92007). The DPLA probe anti-rabbit minus binds to the PTPIP51 antibody, whereas the PLA probe anti-mouse plus binds to the antibody against the probable interaction partner (see Table 1), respectively. The DuoLink proximity ligation assay secondary antibodies generate only a signal when the two DPLA probes have bound, which only takes place if both proteins are closer than 40 nm, indicating their interaction [35]. PFA-fixed HaCaT cells were pre-incubated with blocking agent for 1 h. After washing in PBS for 10 min, primary PTPIP51 antibody (1:1000) was applied to the samples. Primary antibodies of the interacting partner (Table 1) were used for proving the interaction by co-incubation with the PTPIP51 antibody. Incubation was done overnight in a pre-heated humidity chamber. Slides were washed three times in PBS for 10 min. Duolink II PLA probes detecting rabbit or mouse antibodies were diluted in the blocking agent in a concentration of 1:5 and applied to the slides followed by incubation for 1 h in a pre-heated humidity chamber at 37 °C. Unbound DPLA probes were removed by washing two times in PBS for 5 min. The samples were incubated with the ligation solution consisting of Duolink II Ligation stock (1:5) and Duolink Ligase (1:40) diluted in high purity water for 30 min at 37 °C. After ligation the Duolink Amplification and Detection stock, diluted 1:5 by the addition of polymerase (1:80), was applied to the slides for 100 min. Afterwards the slides were incubated with Dapi for the identification of nuclei. After the final washing steps the slides were dried and cover slips were applied.

Quantification was done with the DuoLink Image Tool (Olink Bioscience, Uppsala, Sweden, v1.0.1.2). The software identifies Dapi positive nuclei for the cell count. Cell borders were set according to the software calculated cell shape using a user defined cell diameter preset. Fluorescence dots of the DPLA were counted for each single marked cell by the software. The signal threshold was adjusted to 135 and the pixel size for spot detection to 5 pixels for each picture.

### 4.10. Statistical Analysis

The quantified DPLA spots were calculated per cell (number of dots/cell) for each picture. The results were standardized to the number of interactions/100 cells. The values were subsequently analyzed by GraphPad Prism 6 using the Dunnett’s multiple comparisons test. Results were considered as significant with *p* < 0.05.

## 5. Conclusions

In the current study we show a new relationship of PTPIP51 within the NFκB signaling pathway, especially the interaction with RelA. Furthermore, we could show that both PTPIP51 mRNA and protein expression levels are regulated by TNFα. The interaction levels of the new established interaction partners of PTPIP51, RelA and IκBα also exhibited a TNFα dose dependency.

To sum up, we assumed that, (1) PTPIP51 expression is regulated by RelA after TNFα stimulation; (2) the complex of PTPIP51/RelA/IκBa has a regulatory function on all complexed proteins; and (3) that PTPIP51 probably links the NFκB signaling to the MAPK pathway in cooperation with RelA.
